# Characteristics of annular surface dielectric barrier discharge with microsecond pulse under water-covered condition

**DOI:** 10.1371/journal.pone.0287773

**Published:** 2024-01-18

**Authors:** Yaozong Xu, Yundong Lai, Junting Qin, Ziyi Gong

**Affiliations:** State Key Laboratory of Power Transmission Equipment & System Security and New Technology, Chongqing University, Chongqing, China; University of Sharjah, UNITED ARAB EMIRATES

## Abstract

Surface dielectric barrier discharge (SDBD) has wide applications in flow control, wastewater treatment, and biomedicine. The dielectric surface of an SDBD actuator is generally attached to the water droplets during applications. Thus far, only a few studies have been conducted on the effects of water covering the dielectric surface on the discharge characteristics of SDBD. Therefore, the effects of water droplets on the discharge of an SDBD actuator based on a repetitive microsecond pulse power supply were investigated in this study. The results show that a filament micro-discharge channel forms between the light and dark regions at the internal edge of the SDBD high-voltage electrode and develops toward the center of the dielectric surface in the region without water droplet coverage. SDBD in the water-covered region was divided into two stages. This paper compares the electrical characteristics of SDBD with and without water droplet, and explores the electric field distortion effect of water droplet endpoints through 3D simulation.Based on the theories of water droplet polarization and gas discharge, the effects of water droplets on plasma development and surface charge accumulation under water-covered condition were analyzed. The water droplet plays a similar role as a "secondary electrode" during the discharge process.

## 1. Introduction

Surface dielectric barrier discharge (SDBD) is a typical atmospheric discharge form that has potential applications in flow control, wastewater treatment, and biomedicine [1–5]. Generally, SDBD often works in water-containing environments. For example, in the field of flow control, SDBD actuators are attached to the surface of aircraft and high-speed trains to control the airflow distribution in the boundary layer [6, 7]. However, in practical applications, aircraft and high-speed trains are generally operated under rainy and windy conditions. Under such conditions, the supercooled water droplets hit the surface of the airframe,Therefore, water droplets often adhere to the surface of SDBD. In the fields of wastewater treatment and biomedicine, research on SDBD mostly focuses on the permeation of plasma and aqueous solutions [8, 9], which results in high humidity in the SDBD working environment. Under the effect of electromagnetic, local thermal, flow, or force fields and other physical fields, physical phenomena, such as droplet splashing and interface turbulence, occur in aqueous solution [10]. Therefore, the dielectric surface is generally covered with water. In the above research, scholars only focused on the application effect of SDBD, and there is almost no in-depth report on the impact of water droplets adhering to the surface of SDBD on discharge.

At present, there is little research on water discharge both domestically and internationally. Research in aqueous working environments mainly focuses on the plasma and high-voltage fields. The field of plasma is focused on anti-icing on the surface of SDBD aircraft; The high-voltage field focuses on the surface flashover of composite insulators with water covering. Considering the research in the field of anti-icing and de-icing of aircraft surfaces, Tirumala et al. [11] found that SDBD can achieve a de-icing effect through thermal effects induced by infrared thermal imaging. Meng and Hu [12, 13] found that the heat accumulation generated by SDBD can cause the surface temperature of the SDBD actuator to reach approximately 15°C. Supercooled water droplets can be effectively heated to form a water film on the airfoil surface. The SDBD produces more heat. Zhu et al. [14] found that the water content is a key factor affecting the icing performance. The discharge current and total energy deposition increased by 3% and 6.7%, respectively, in the presence of water on the SDBD surface. The anti-icing ability became stronger with an increase in the water content.Liu et al. and Wei et al. [15, 16] found that the instantaneous value of the discharge voltage and the oscillation of the current curve decrease when water drop-lets strike the surface of the SDBD actuator. Zheng et al. [17] found that when droplets collide with an SDBD surface, the air and electrodes are fully protected by water. Therefore, the discharge was inhibited, and the inhibition area gradually expanded with increasing discharge time. Peng et al. [18] observed that the discharge intensity was significantly enhanced when water was attached to the surface of a three-electrode SDBD system. In anti-icing and de-icing research, it has been assumed that aircraft wings are subjected to icing, and more studies have focused on the thermal de-icing effect of the SDBD. However, these studies did not consider the impact of water on discharge during the process of converting water to ice. Several studies have demonstrated that water droplets can affect the discharge characteristics of SDBD; however, in-depth studies on this phenomenon have not been conducted.Previous studies in the field of anti-icing have only focused on the impact of SDBD on ice. The effect of water-covered dielectric surfaces on discharge has not been sufficiently explored. Currently, most applications of SDBD under water-covered conditions are based on the effects of discharge on water. However, under water-covered conditions, water has an impact on the discharge. For example, when water droplets cover an insulator surface, they distort the electric field distribution on the dielectric surface in a wet environment [19, 20]. Partial discharge occurs around water droplets and causes flashover [21–23]. Therefore, studying the discharge characteristics of SDBD under water-covered conditions is important for its application in these fields.

Numerous studies have been performed on strip electrode SDBD for SDBD applications. Our group demonstrated that annular SDBD can generate vertical flow fields and have a high airflow altitude [24], indicating good application prospects in aircraft anti-icing [25]. Therefore, based on our previous research results on the discharge characteristics of SDBD actuators [2, 3, 25, 26], this study focused on the discharge characteristics of annular SDBD under water-covered conditions. In this study, the characteristics of the discharge images were studied using image grayscale processing. Furthermore, the external electric field distribution of the annular SDBD under water-covered conditions were calculated by simulation modeling, and by combining it with gas discharge theory, the plasma development process and the influence of water droplets on the discharge of the annular SDBD under water-covered conditions were analyzed.

## 2. Experimental equipment and measurement system

**Fig 1:**
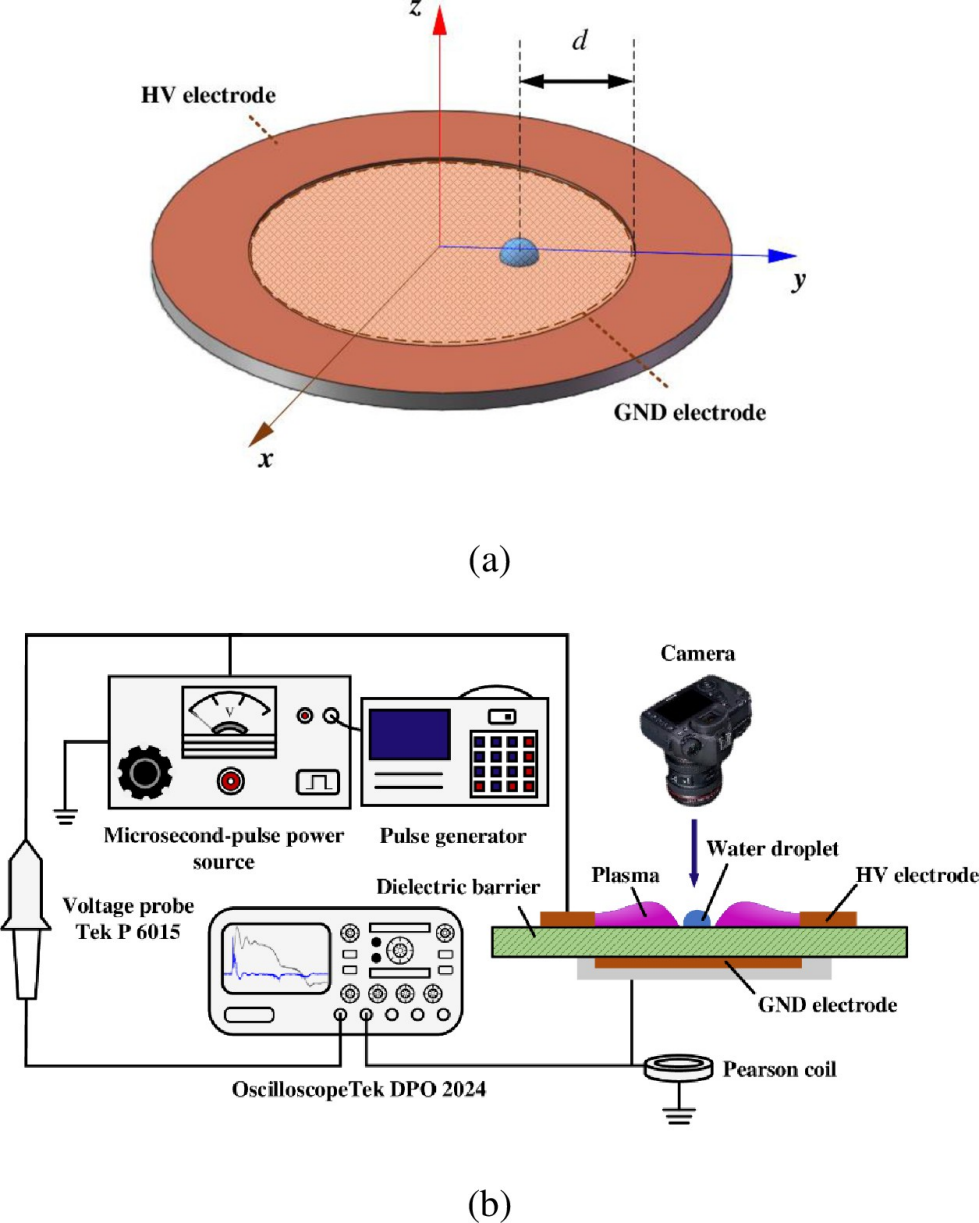
Annular SDBD under water-covered condition: (a) Schematic diagram of SDBD structure under water-covered condition, (b) Experimental equipment and measuring system.

The experimental equipment and measurement system for the annular SDBD under water-covered conditions are shown in Fig 1. All discharge experiments mentioned in this paper were conducted under atmospheric-pressure air conditions. The epoxy barrier dielectric of the annular SDBD actuator used in this study possessed a relative dielectric permittivity of 2.4 and thickness of 1 mm. The high-voltage and ground electrodes were placed on the upper and lower sides of the dielectric barrier in two concentric circles, respectively. The horizontal distance between the internal edge of the high-voltage electrode and external edge of the ground electrode was 0 mm. The thickness of the high-voltage and ground electrodes was 35 μm, and the width of the copper annular high-voltage electrode was 5 mm. The internal edge of the high-voltage electrode was directly opposite to the external edge of the ground electrode. The radius of the internal annular edge of the high-voltage electrode is the internal radius, *r*, and the values of *r* and radius of the ground electrode are both 10 mm. Plasma develops from the internal edge of the high-voltage electrode to the center of the dielectric ring; this is known as an inward discharge. The ground electrode was encapsulated in silica gel to avoid surface flashover [26, 27]. In the experiment, the volume of each water droplet was controlled to 1 μL using a micro- sampler with a total capacity of 50 μL. The water droplet on the surface of the dielectric was hemispherical in shape with an average radius of 0.75 mm, which was consistent with the calculated value. In this study, the distance between the center of the water droplet and the internal edge of the high-voltage electrode is defined as *d* as shown in Fig 1(A).

The experiment was conducted using the repetition frequency microsecond pulse power supply CMPC-40D developed by the Institute of Electrical Engineering, Chinese Academy of Sciences. Its output voltage was up to 30 kV, and the repetition frequency varied from 0 to 3 kHz, with a rising edge of 500 ns. In our previous study, we demonstrated that the repetitive frequency had negligible effects on the discharge characteristics of annular SDBD[3, 28]; therefore, we maintained the repetitive frequency at 800 Hz in the experiments. The applied voltage was measured using a high-voltage probe (Tek P6015A). The current was measured using a Pearson Model 4100 instrument. An oscilloscope (Tektronix DPO2024) was used to record voltage and current signals. Discharge images were captured using a Canon EOS 5DIII digital camera with a macro lens CA-Dreamer Macro 2X 100 mm F2.8. The exposure time T, sensitivity ISO, and aperture F of the camera significantly affect the capture of the discharge image. The exposure time was used to measure the intensity of light blocked by the shutter in front of the lens. The light intake of the lens was proportional to the exposure time T. The International Standard Organization ISO was employed to adjust the sensitivity of the photosensitive devices. Aperture F was used to adjust the amount of light in the lens. The light input of the lens is proportional to the exposure time T and sensitivity ISO and inversely proportional to the aperture F. The camera exposure time T and sensitivity ISO were set to 1 s and 1600 and 1/800 s and 52,100, respectively, to obtain multi-pulse superposition and single-pulse discharge images with an aperture F = 4.0.

## 3. Results

### 3.1 Typical discharge characteristics

**Fig 2:**
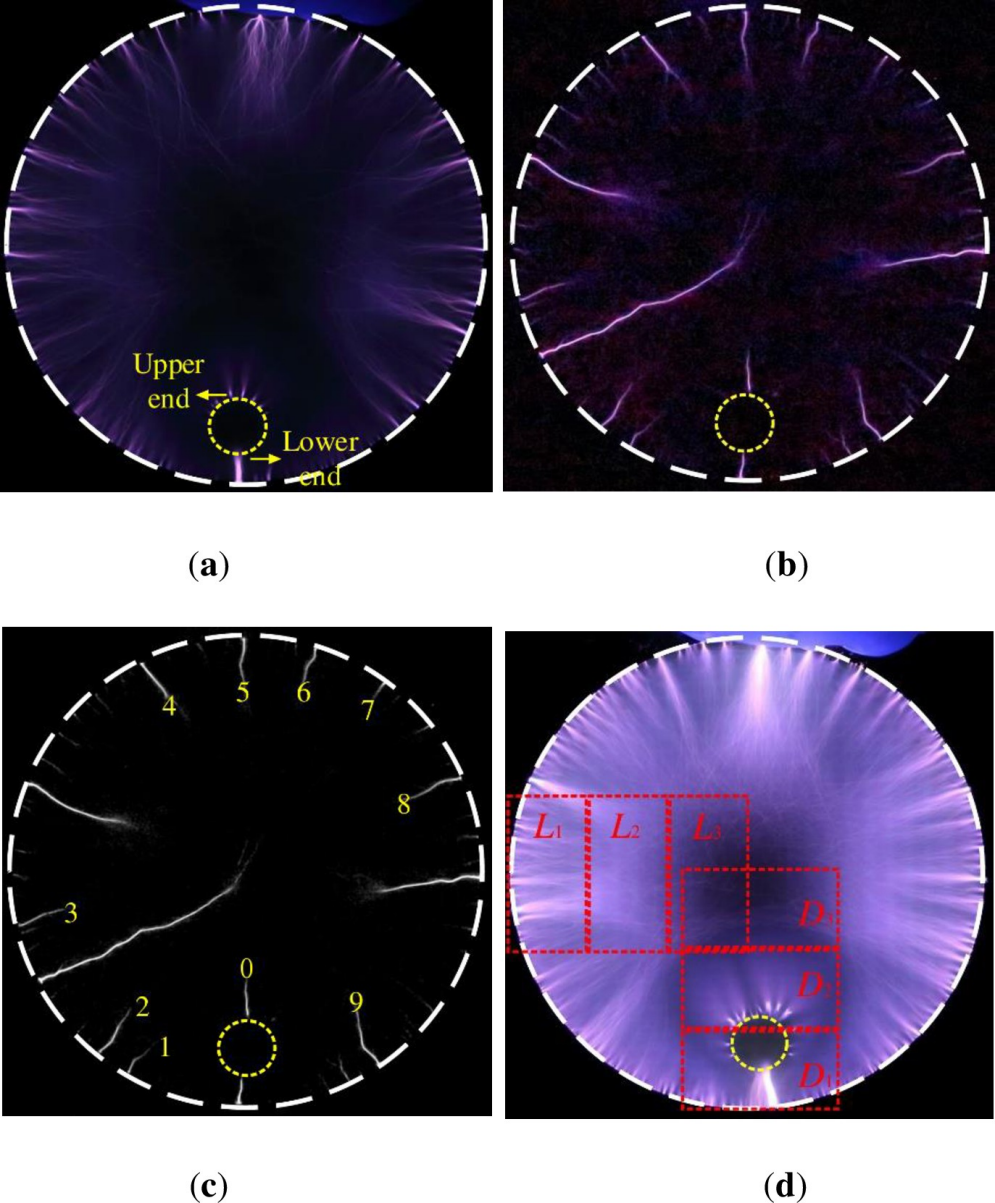
Typical discharge image (d = 2 mm): (a) 100 pulse superimposed discharge images, (b) Single pulse discharge image, (c) Discrete micro-discharge channels 0~9 processed by gray scale in single pulse discharge image, (d) Different regions in 800 pulse discharge image.

A typical discharge image of an annular SDBD under water-covered condition is shown in Fig 2, where the amplitude of the applied voltage is 16 kV and *d* = 2 mm. Fig 2(A) and 2(B) show a 100-pulse superimposed discharge image and a single-pulse discharge image, respectively. The internal edge of the high-voltage electrode and the water-droplet-covered placement area are marked with white and yellow dotted lines, respectively. In this study, the lower edge of the water droplet near the high-voltage electrode was defined as the lower end of the water droplet, and the edge of the other side of the water droplet was defined as the upper end of the water droplet, which was replaced with the lower end and upper end in the following. The discharge is generated at the internal edge of the high-voltage electrode and points to the center of the dielectric ring when an external voltage of 16 kV is applied in the water droplet-uncovered region, as shown in Fig 2(A). The discharge mainly consists of bright discrete micro-discharge channels, and a clear bifurcation phenomenon is observed during the development of the micro-discharge channel. The head of the channel is distributed in a diffusive shape. This is similar to the results of Li et al. [25].

The discharge in the water-droplet-covered area is divided into two stages. In the first stage, a micro-discharge channel is generated from the internal edge of the high-voltage electrode. Subsequently, a micro-discharge channel develops near the lower end along the dielectric surface. The micro-discharge channel has a stronger luminous intensity than other areas and finally bridges with the water droplet. However, realizing a contact between the adjacent micro-discharge channel around the high-voltage electrode at the lower end with the water drop is difficult, and the discharge is relatively weak. This is because during the discharge process, when a micro-discharge channel develops rapidly and violently, the charge density and conductivity in the channel are high, thereby inhibiting the formation and development of the surrounding micro-discharge channel [29]. A significant discharge is not observed in the area covered by the water droplets.

In the second stage, a micro-discharge channel is formed at the upper end and extends along the dielectric ring to the center. The morphology of the micro-discharge channel formed at the upper end is similar to the outward discharge morphology of the SDBD [2]. The micro-discharge channels formed at the upper end are mainly discrete channels. For single-pulse exposure, the diffuse discharge phenomenon is evident at the root of the micro-discharge channel, whereas it occurs dispersion at the front of the channel, as shown in Fig 2(B).

**Fig 3:**
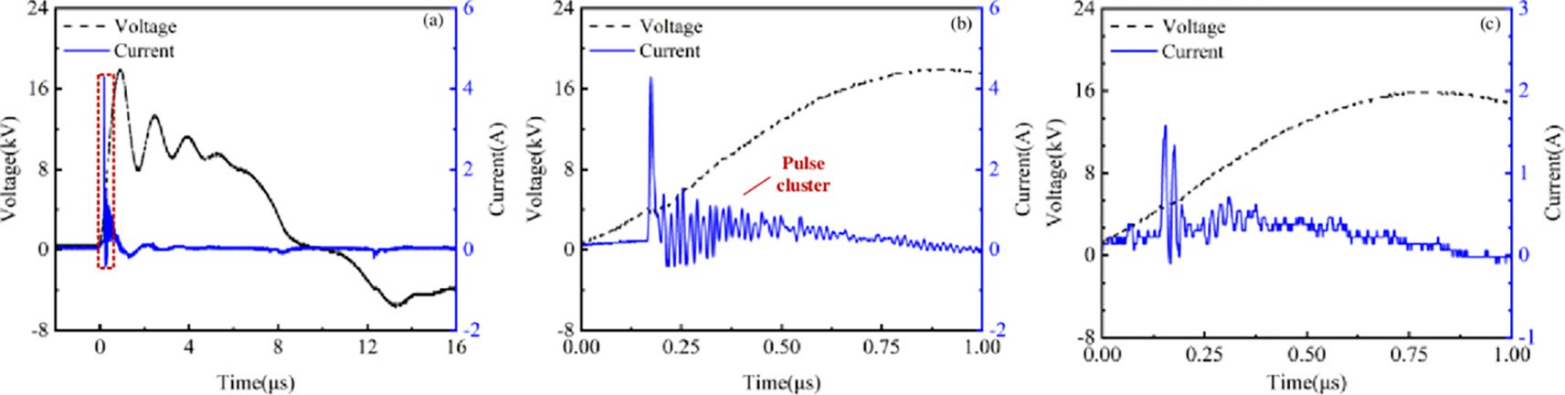
Typical current waveform of annular SDBD under no water-covered and water-covered condition (*d* = 2 mm).

The typical discharge-current waveform of annular SDBD under no water-covered condition is shown in Fig 3(A). Current discharge occurred at the rising edge of the voltage under pulse excitation. The current waveform rapidly reaches its maximum value and has multiple peaks, which are consistent with the results obtained by Li et al. [28].The current waveforms within 1 μs under no water-covered condition and water-covered condition are shown in respectively Fig 3(B) and 3(C). The current waveform is in a multi-pulse cluster combination state because the voltage waveform of the microsecond pulse power supply is not changed by a single peak value. It can be found that the amplitude of the annular SDBD current significantly decreases under the water-covered condition through comparison of Fig 3(B) and 3(C).Additionally, the position of the current pulse peak can be clearly distinguished in Fig 3. The current pulse peak had a short duration and then began to oscillate and decline, gradually decaying to 0 A. The duration of current peak is shorter and the number of current drop pulse clusters is significantly less under water-covered condition than that of the absence of the water droplet. Two factors affected the duration of the current pulse peak. On the one hand, the discharge leads to the accumulation of charges on the surface of the annular dielectric, In contrast, the micro-discharge channels develop at the lower end blocked the movement of the charges. A large number of charges accumulate at the end under water-covered conditions during discharge. Therefore, the charges on the dielectric surface are superimposed on each other, rendering it more difficult to generate a new discharge channel each time, as indicated by the duration of the pulse peak on the current waveform. Under water-covered condition, the positive charge at the head of the micro discharge channel is more likely to accumulate at the lower end and slow down as the channel develops, resulting in a rapid decrease in the gap voltage. The discharge channel generated at the inner edge of the high-voltage electrode around the water droplet is greatly suppressed, making it difficult to generate a new micro discharge channel between the high-voltage electrode and the lower end. Therefore, the current waveform is more dispersed and the number of pulse clusters is small during the oscillation and decrease process.

**Fig 4:**
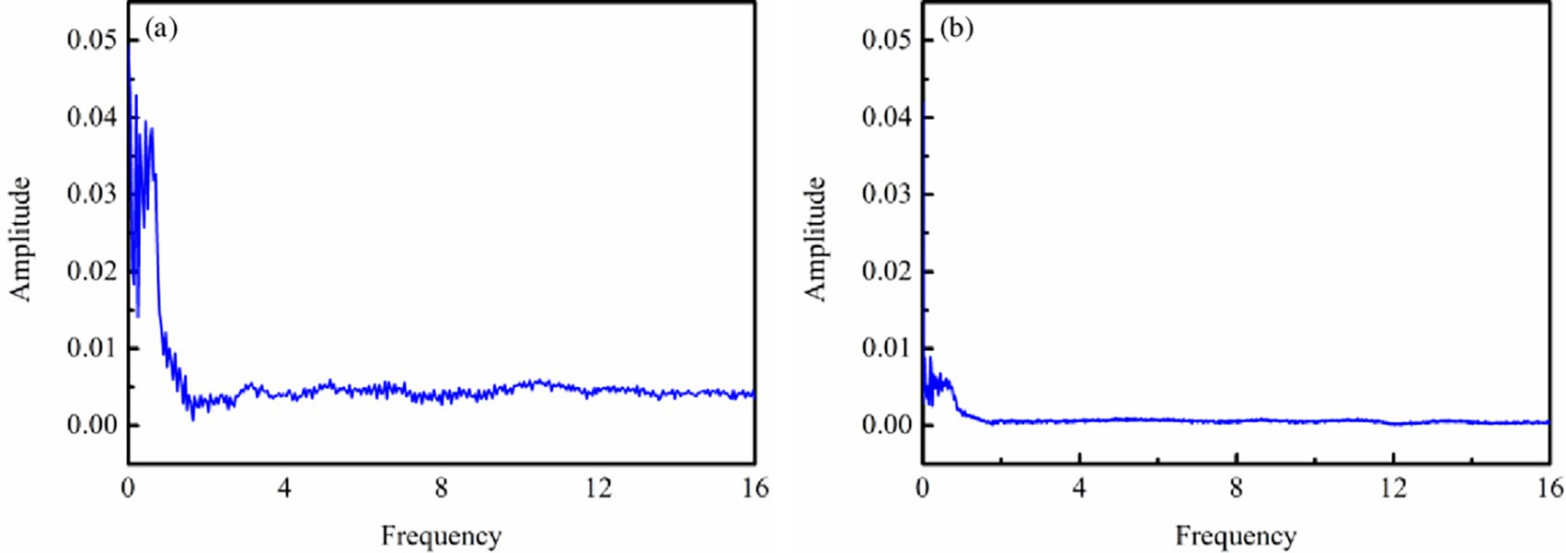
Fast Fourier transform of current of annular SDBD under no water-covered and water-covered condition.

SDBD is capacitive in static state, but different from general capacitive loads. Its discharge circuit includes an air gap and a dielectric barrier layer, and its equivalent circuit is two capacitive loads connected in series. The total dynamic equivalent circuit capacitance of SDBD undergoes significant changes during discharge and non-discharge, with the air gap capacitance being broken down. When discharge occurs, the total equivalent capacitance suddenly increases by several times or more than before discharge. Therefore, SDBD always generates electromagnetic interference when working under high voltage conditions. The annular SDBD current absence of the water droplet and in the presence of a single water droplet were presented the Fast Fourier Transform (FFT) as shown in Fig 4(A) and 4(B), respectively. It can be found that after being covered with water, harmonics reduces in the current through comparison. This indicates that the presence of water droplet serves as current limiting protection, which will suppress high-frequency clutter interference from the power supply side. Thus, the electromagnetic interference generated by the plasma actuator decreased under water-covered condition. This is similar to the research results of Hou et al. [30].

The collected discharge images were RGB true-color images captured by the camera during the experiments. However, Li et al. [31] suggested that the intensity of the discharge can be reflected in the grayscale image when air is used as the working gas. Therefore, to study the overall characteristics of the discharge, such as the intensity and uniformity of the discharge, under water-covered condition, the discharge images were processed into grayscale images through digital image processing. Using the grayscale information of the SDBD images, the image contour, brightness level, and other characteristic information can be obtained from the grayscale images, and the results are is consistent with those obtained using the true RGB color images. The gray value of the grayscale images can be used to indicate the brightness of the discharge. The mean and standard deviation of the gray values are important parameters for describing grayscale images [32, 33]. In this study, *M* and *Std* represent the mean and standard deviation of the gray values, respectively, and were calculated using Equs 1 and 2, respectively.

$$M=\bar{G_{1}(x,y)}=\frac{1}{n•m}\sum_{j=1}^{n}\sum_{i=1}^{m}G_{1}(x_{i},y_{j})$$ (1)

$$StdDev=\sqrt{\frac{1}{n•m}\sum_{j=1}^{n}\sum_{i=1}^{m}{\left[G_{1}(x_{i},y_{j})-\bar{G_{1}(x,y)}\right]}^{2}}$$ (2)

where *n* and *m* are the numbers of pixels in the *x* and *y* directions of the discharge area, respectively. *M* is generally used to describe the overall distribution of the gray images and reflects the discharge intensity. *Std* reflects the degree of dispersion of the gray intensity of the discharge image compared with *M*. When *Std* is small, the differences between the gray values are small, and the gray intensity is closer to *M*. This shows that the gray intensity distribution in the gray image is more uniform, which indicates that the distribution of the micro-discharge channels in the discharge image is also more uniform.

**Fig 5:**
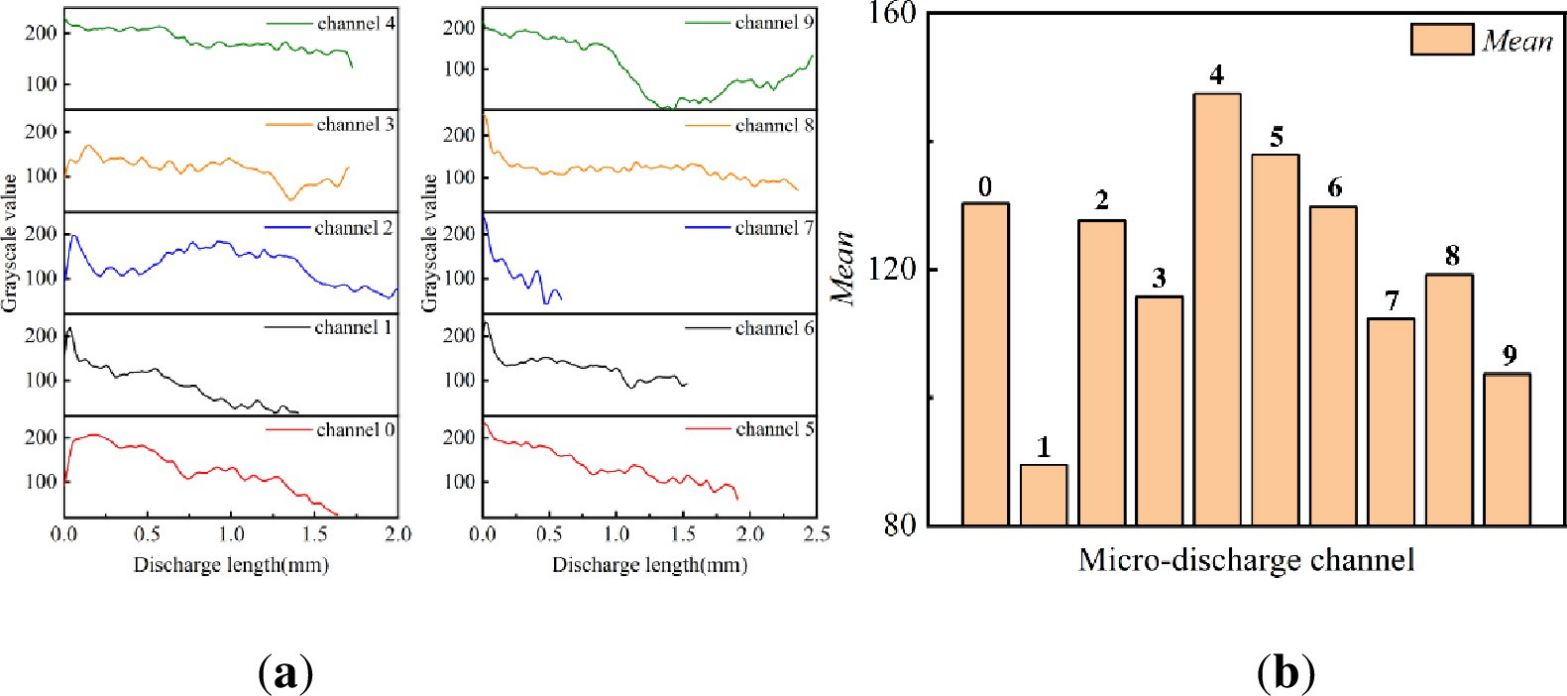
Gray value change of micro-discharge channels in single-pulse discharge image: (**a**) Grayscale curve of micro-discharge channels in single-pulse discharge image, (**b**) Mean of micro-discharge channels gray value of single pulse discharge image.

The discrete micro-discharge channels 0–9 of the single-pulse discharge image are processed in grayscale, as shown in Fig 2(C). Micro-discharge channel 0 is generated from the upper end, whereas micro-discharge channels 1–9 are generated from the inner edge of the high-voltage electrode. The width and length of the discrete micro-discharge channels around the high-voltage electrode and at the upper end are calculated using the pixel points, and a gray intensity curve is drawn along the discrete channels to show the intensity of the micro-discharge channels, as shown in Fig 5.

The width of the channels 0–9 is approximately 0.09 mm as calculated using pixel points in Fig 2(C). The length of these discrete channels is between 0.6 and 2.5 mm, as shown in Fig 5(A). Li et al. [31] also obtained actual discharge data using pixel points while studying corona discharge RGB digital images. The length of the micro-discharge channel at the upper end is 1.64 mm, which is within 0.6–3.5 mm. This is similar to the conclusion obtained in a microsecond-pulse SDBD study by Li [28]. The gray values of micro-discharge channels 0–9 decrease with an increase in the discharge channel length. This numerically proves that the discrete channels are generated both from the inner edge of the high-voltage electrode and from the upper end; the discharge is more intense at the root of the channels, and the intensity gradually decreases with the development of the channels. The mean gray values of the discrete channels in the single-pulse discharge image are shown in Fig 5(B). The discharge gray value of channels 1–9 is between 89.5 and 147.4, and the average gray value of channel 0 is 130.2. This implies that the micro-discharge channel formed at the upper end is similar to the discrete micro-discharge channels generated at the inner edge of the high-voltage electrode in terms of width, length, and discharge intensity.

In the 800-pulse superimposed discharge image, three rectangular regions of 3 mm × 6 mm captured along the radial direction of the left and lower sides of the annular SDBD are shown in Fig 2(D). These rectangular areas were processed in grayscale. The different M and Std values for each region are listed in Table 1.

**Table 1:** Values of *M* and *Std* in each area.

Region	*Mean*	*StdDev*
*D*_1_	117.65	51.87
*D*_2_	104.89	28.45
*D*_3_	72.37	23.93
*L*_1_	170.26	38.96
*L*_2_	156.41	16.10
*L*_3_	90.58	14.27

Comparing regions *L*_1_ and *D*_1_, the mean value *M* of the region without water droplets is significantly higher than that of the region covered with water droplets, whereas *std* is slightly smaller. This shows that the overall discharge intensity in region *D*_1_ is lower than that in *L*_1_, and the degree of dispersion of the discharge is large with low uniformity. Comparing regions *L*_1_ to *L*_3_, *M* and *Std* gradually decrease with the development of the micro-discharge channels. The discharge intensity gradually decreases with the development of the channels, and the discharge uniformity gradually improves. This indicates that the SDBD gradually transitions to a mixed discrete diffusion mode. For *D*_1_–*D*_3_, the change trends of *M* and *Std* are similar to those of *L*_1_–*L*_3_. As listed in Table 1, the presence of water droplets weakens the overall intensity of the SDBD and reduces the uniformity of the discharge under water-covered condition.

In this study, data analysis reveals that an independent micro-discharge channel is generated at the upper end and diffused toward the center of the dielectric surface, which is consistent with the observation of the discrete channels generated by the high-voltage electrode. Baroch et al. [34] used the water surface as an electrode when studying wastewater degradation using SDBD. Therefore, this paper considers that water droplet plays a similar role as a "secondary electrode" during SDBD discharge under the water-covered condition. The SDBD discharge is extended, although the uniformity is reduced.

### 3.2 Dynamic deformation of water droplets

**Fig 6:**
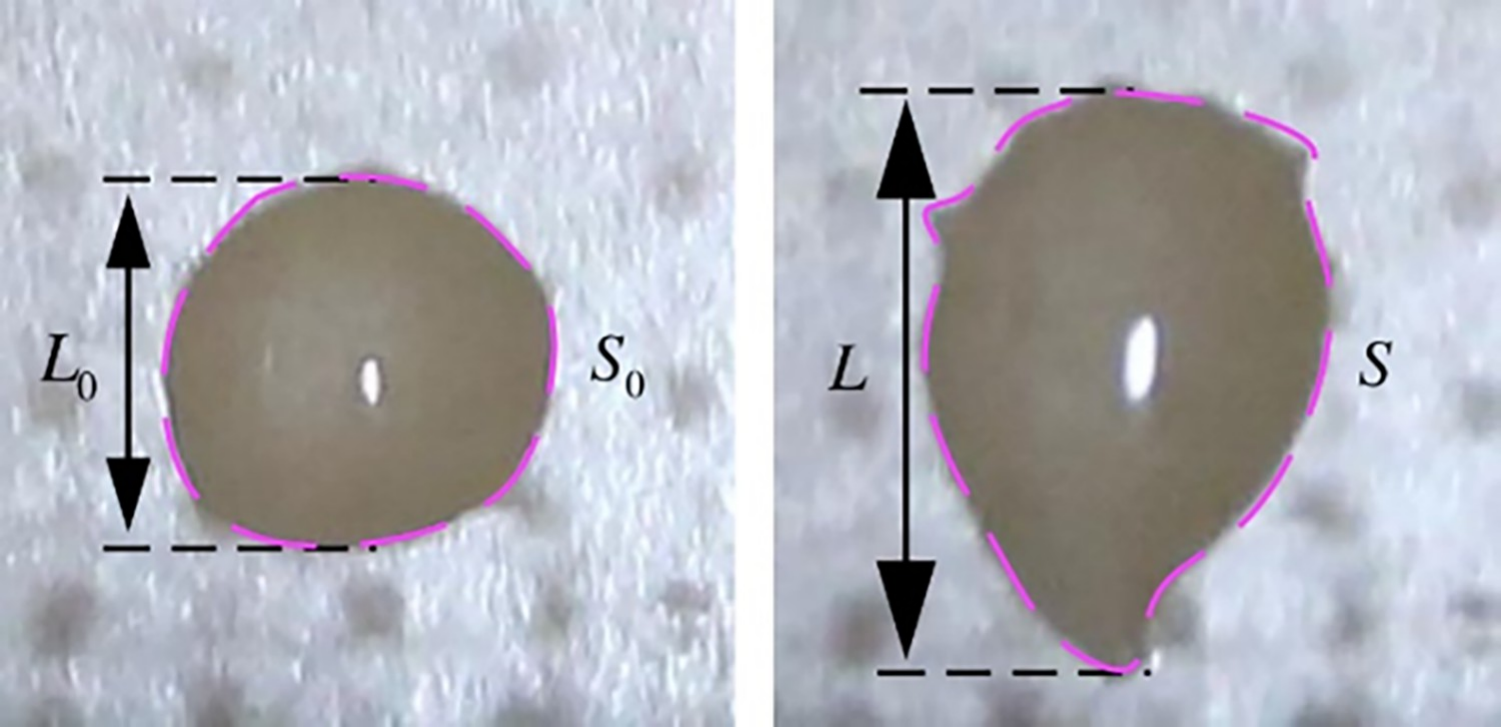
Diagram of water droplet deformation after the discharge of SDBD.

This article defines the distance from the center of the water droplet to the inner edge of the high-voltage electrode as *d*. Discharge experiments were conducted by changing the distance of *d*. Hemispherical water droplet will vibrate under the action of electric field force, and continuous vibration causes the water droplets to deform along the direction of electric field force, resulting in the formation of flat and sharp tip edges at the boundary of gas, liquid, and solid phases. The water droplets no longer maintain a hemispherical shape, but adhere to the dielectric surface in an irregular form. Taking the image of a water droplet with a voltage amplitude of 16 kV and *d* = 10 mm after discharge as an example, the influence of SDBD discharge heating effect on the water droplet is ignored. The initial hemispherical diameter of the water droplet *L*_0_ = 1.52 mm and the hemispherical bottom area *S*_0_ = 1.81 mm^2^ were calculated through image processing. The actual data is basically consistent with the theoretical values. After the discharge, the hemispherical shape of the water droplet is gradually stretched into a semi ellipsoidal shape, with a maximum length of *L* = 2.5 mm. The water droplet is elongated by about 60%, and the bottom area of the semi ellipsoid *S* = 2.65 mm^2^. Through calculation, the height of the semi ellipsoidal water droplet after discharge *H* = 0.48 mm, and the height of the water droplet is reduced by about 36%. For quantitatively analysis of the dynamic behavior of water droplet deformation, this section defines the deformation factor K based on the initial length and height of the water droplet, as well as the length and height of the water droplet after discharge. The parameter diagram of the water droplet shape variable is shown in Fig 6.

In Fig 6, *L*_0_ is the initial length of the water droplet, and *L* is the length of the water droplet after discharge deformation; *S*_0_ is the hemispherical bottom area of the water droplet, and *S* is the semi ellipsoidal bottom area of the water droplet after discharge deformation. Define the initial height of the water droplet *H*_0_ = *L*_0_, and the semi ellipsoidal height of the water droplet after deformation is *H*. Define *M*_0_ = *L*_0_/*H*_0_ as the initial water droplet state factor, and the initial water droplet is a hemispherical water droplet. Therefore, *L*_0_ = *H*_0_, *M*_0_ = 1, *M* = *L*/*H* is the water droplet state factors after discharge deformation. Therefore, the definition of the deformation factor *K* of the water droplet is shown in Eq 3.


$$K=\frac{M}{M_{0}}=M$$ (3)

**Fig 7:**
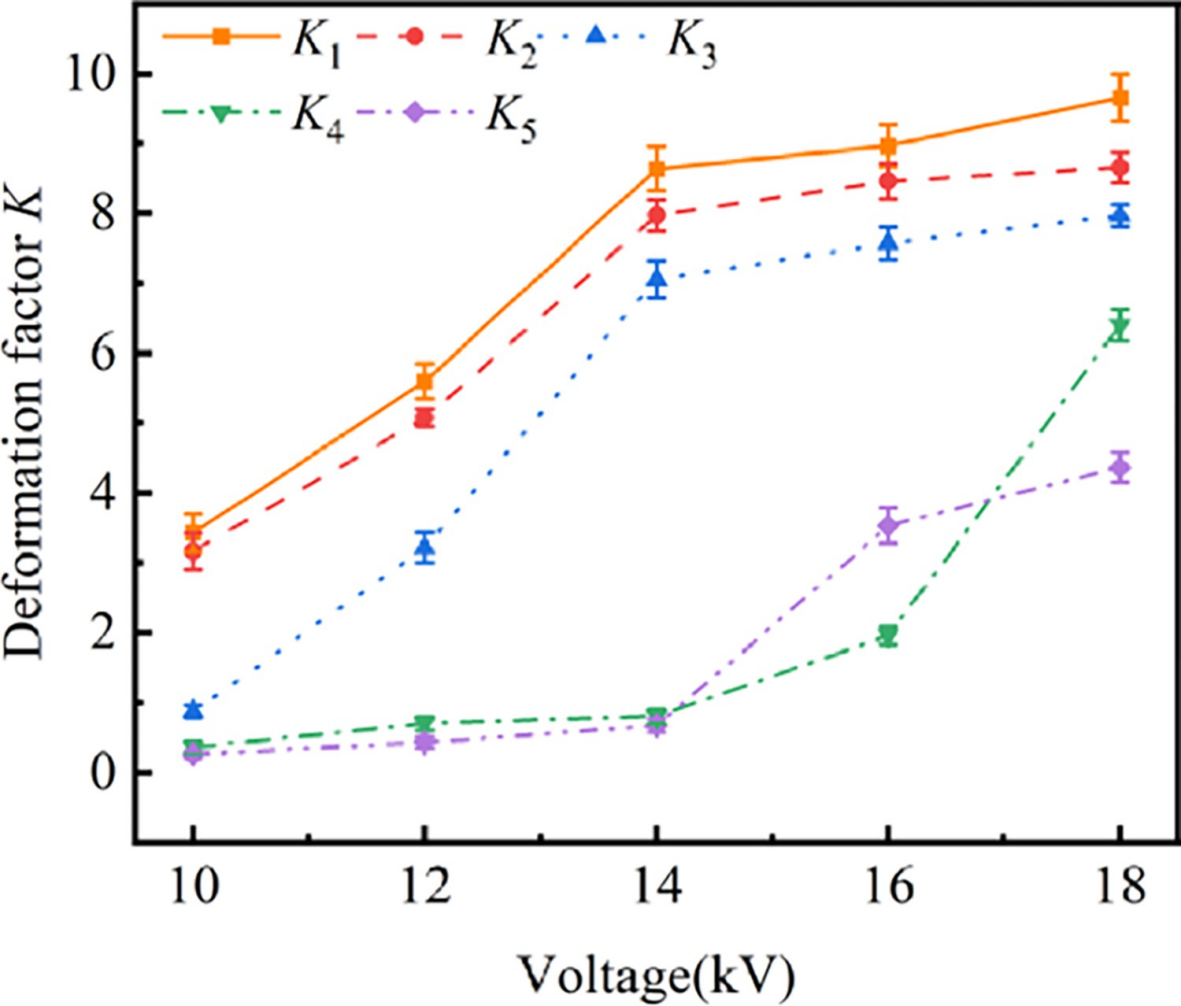
Deformation factor *K* of water droplet at different voltage amplitudes and water-covered positions.

The variation of droplet deformation factor K after discharge under different voltage amplitudes and droplet attachment positions is shown in Fig 7. The plot K1, K2, K3, K4, and K5 correspond to the K of positions d of 2 mm, 4 mm, 6 mm, 8 mm, and 10 mm, respectively. As shown in Fig 7, with the increase of voltage amplitude, K under different d shows an increasing trend. When d is less than 8 mm, the maximum slope of the deformation factor increase occurs at the voltage from 12 kV to 14 kV, which is also due to the increase in the intensity of the discharge at this time. The discharge gradually presents a mixed mode of dispersion and dispersion. When d is 8 mm and 10 mm, K approaches 0 before 14 kV, because the micro discharge channel has not yet developed to the water droplet when the voltage amplitude is less than 14 kV. When the voltage continues to rise, the micro discharge channel develops to the water droplet, and the water droplet rapidly deforms. Overall, the deformation factor K increases with the increase of voltage amplitude and decreases with water position d away from the high-voltage electrode.

### 3.3 Effect of droplet properties on discharge

Attach insulating oil, 75% alcohol, distilled water, tap water, and saturated salt water to the surface of the annular SDBD to explore the effect of conductivity on the discharge characteristics. The conductivity of different droplets is shown in Table 2.

**Table 2:** Conductivity parameter of different droplet.

Droplet	Conductivity/μS cm^-1^
insulating oil	5×10^−10^
75% alcohol	2.1×10^−1^
distilled water	20
tap water	675
saturated salt water	2.51×10^5^

**Fig 8:**
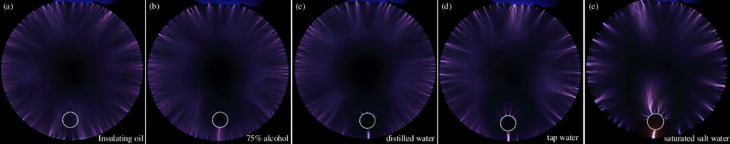
Discharge images of droplets with different conductivity.

Discharge images of droplets with different conductivity adhering to the dielectric surface of annular SDBD is shown in Fig 8, where the amplitude of the applied voltage is 16 kV. The white circular dashed line represents the position of the droplet attachment. As the conductivity of the droplet gradually increases, the discharge luminescence intensity in the first stage gradually increases, and the micro discharge channel in the second stage is also more obvious. When saturated saline water is attached to the dielectric surface, the micro discharge channels at the first stage is intense and a red channel is formed at the head, which is similar to the phenomenon of discharge in water [35]. A large number of discrete plasma channels are generated at the upper end of saline droplets, and the micro discharge channels in the second stage gradually become dispersed at the head. This is because there are already many freely moving ions inside the saturated saline water.

**Fig 9:**
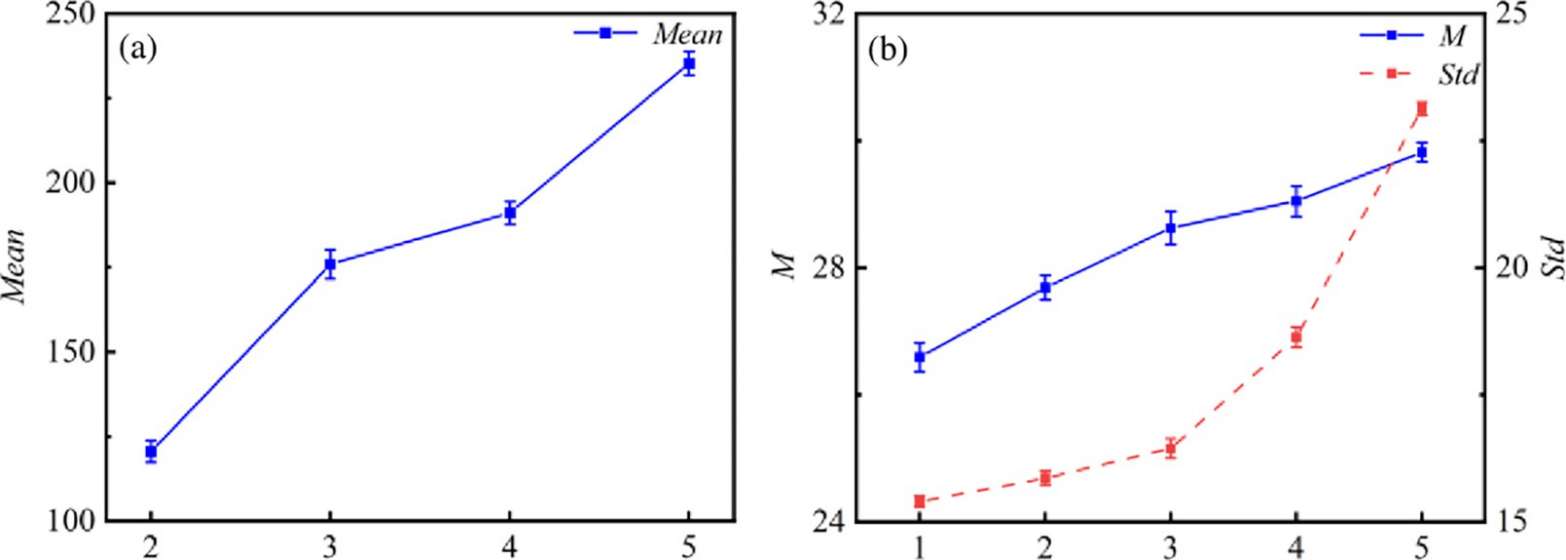
Discharge gray value change under the attachment of droplets with different conductivity: (a) Grayscale intensity *Mean* in the first stage of discharge, (b) *M* and *Std* of overall grayscale values of SDBD.

Number Fig 7(A) to (e) as Figs 1–5. Perform grayscale processing on the discharge image and calculate the mean grayscale intensity of first stage micro discharge channels of 2~5, as well as M and Std of overall SDBD discharge grayscale values of 1~5, as shown in Fig 9. With the increase of droplet conductivity, the grayscale intensity of the first stage of discharge significantly increases, indicating that the intensity of discharge in this stage becomes stronger. The M of the overall discharge of SDBD slightly increases with the increase of droplet conductivity with the Std increasing. This indicates that the discharge intensity increases while the discharge uniformity decreases and the discharge of SDBD is mainly in discrete channels with the increase of droplet conductivity. The droplet with high conductivity leads to more frequent collisions of electrons, and the charge spreads more easily on the surface diffusion of the dielectric and accumulates around the surface and droplet. The electric field at the head of the plasma channel gets a faster compensation effect, so a discrete channel with higher electron concentration and shorter length will be generated.

**Fig 10:**
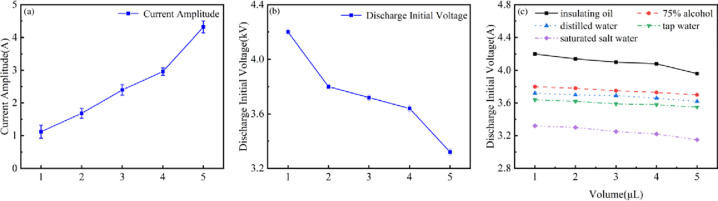
Variation of current amplitude and discharge initial voltage: (a) Changes in current amplitude under different conductivity, (b) Changes in discharge initial voltage with the same volume under different conductivity, (c) Changes in discharge initial voltage for different volumes with the same conductivity.

In this article, five different droplets were labeled in the order of conductivity from small to large as 1~5. The changes in current amplitude under different conductivity droplet attachment are shown in Fig 10(A). As the droplet conductivity increases, the pulse amplitude of the discharge current significantly increases. This is because the magnitude of conductivity indicates the strength of the current conduction ability of different droplets, and higher conductivity represents higher ion concentration and mobility. Therefore, as the conductivity of droplets increases, ions are more likely to migrate on the dielectric surface, resulting in a larger current under the high electric field, which is similar to the research results of pulsed discharge in water [36].

At the same volume, with the increase of droplet conductivity, the initial discharge voltage gradually decreases as shown in Fig 10(B), which is similar to the research results of Gasanova [37]. Droplets with higher conductivity are more prone to polarization under the external electric field. More freely moving ions exist on the dielectric surface and around the droplet. The gap electric field between the high-voltage electrode and the droplet is larger, making discharge more likely to occur. In addition, the study also found that under the same conductivity, as the droplet volume increases, the discharge initiation voltage also slightly decreases.

## 4. Discussion

### 4.1 External electric field distribution

The electric field is a very significant parameter for exciting gas discharge. Although the space electric field in the discharge process includes the external electric field generated by the external voltage excitation and the electric field generated by the charged particles, discharge occurs only when the external electric field of the discharge area reaches a certain threshold.[3] The external electric field plays a key role in the discharge. Therefore, an integrated discussion on the distribution of the external electric field is vital for understanding the generation and development of plasma. Considering the discharge plasma and charged particles, the external electric field ***E***_ex_ can be calculated using the Laplace equation:

$$E_{\mathrm{ex}}=-\nabla \varphi$$ (4)

Where ∇ represents the gradient operator, and *φ* represents the scalar potential.

At the interface between air, water, and the dielectric surface, owing to a large dielectric constant, high breakdown field strength, and strong self-recovery ability of water, the water-covered region was set to charge conservation. The relative permittivity *ε* of water was set to 80, and the tangential component of the electric field strength was continuous at the three-phase interface. Therefore, the dielectric interface condition of the electric field strength was defined as follows.


$$E_{1t}=E_{2t}$$ (5)

In the absence of a free surface charge at the dielectric interface, the density of the free surface charge *σ* was set as 0. The normal component of the electric flux is continuous, and the dielectric interface condition of the electric displacement vector was set as follows.


$$D_{1t}=D_{2t}$$ (6)

The conventional two-dimensional cross-section model cannot accurately describe the external electric field distribution of an annular SDBD because of the axisymmetric structure of the annular SDBD electrode [25]. Therefore, a three-dimensional model was built to study the distribution of the external electric field of the annular SDBD under water-covered condition based on Fig 1(A). The applied excitation voltage was 10 kV for the external electric field simulations of the non-water-covered and water-covered annular SDBD. The water-covered position *(d*) was set to 2 mm. A three-dimensional simulation model of the *x*, *y*, and *z* axes was established with the circle center of the annular SDBD as the origin.

**Fig 11:**
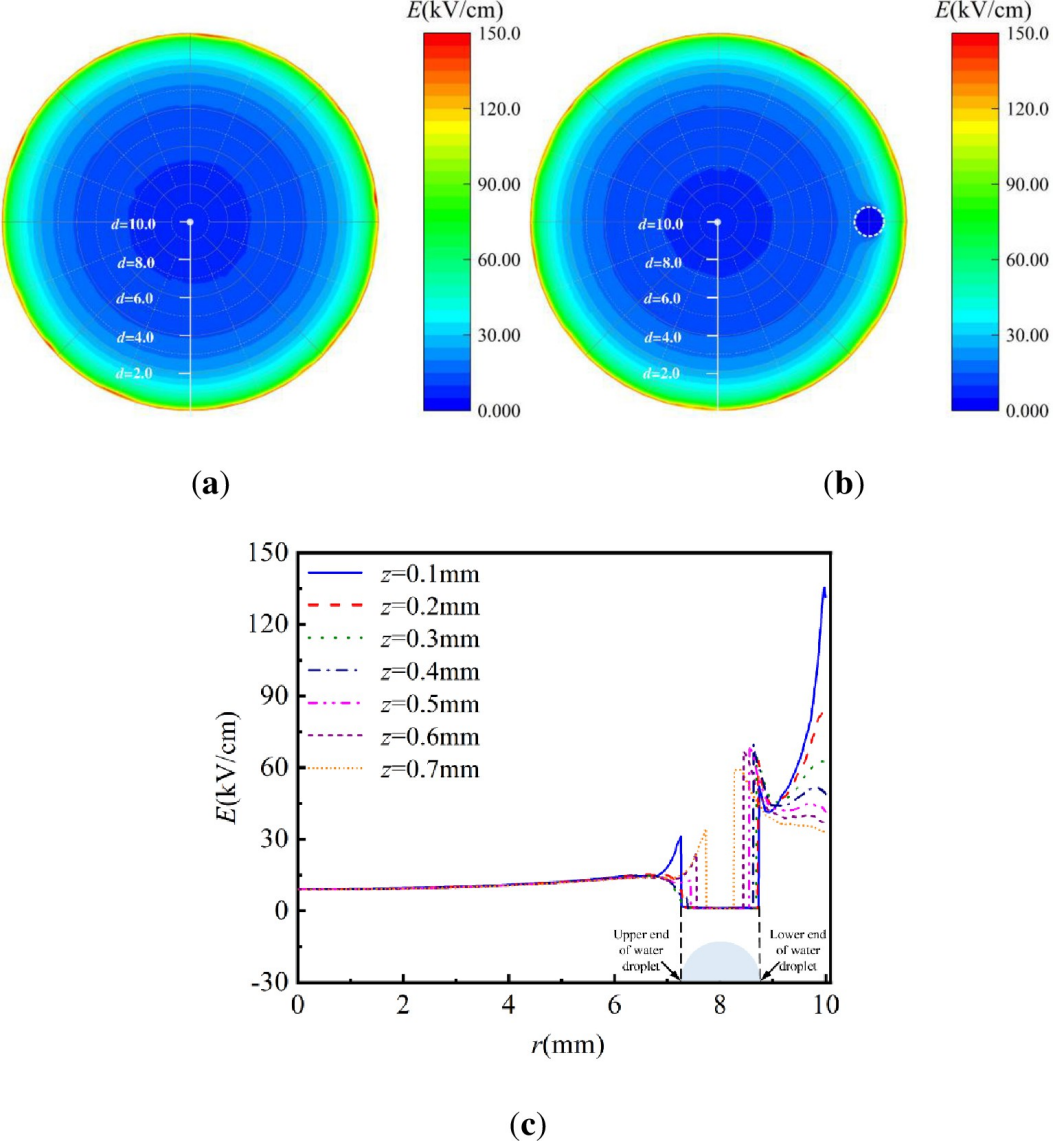
Contour and curve of external electric field of annular SDBD under water covering condition (*d* = 2 mm): (**a**) Contour of annular SDBD electric field under no water-covered condition, (**b**) Contour of annular SDBD electric field under water-covered condition, (**c**)Curves of variation of external electric field with r under different z values.

Fig 11(A) depicts the electric field contour of the internal edge area of the high-voltage electrode with and without water coverage. The white line segment in the figure represents the distance from the center of the annular SDBD to the high-voltage electrode, whereas the attachment position of the water droplet is marked by a white circular dotted line. The high-intensity region of ***E***_ex_ is concentrated near the internal edge of the high-voltage electrode, confirming that the external electric field intensity, ***E***_ex_, plays a significant role in the generation and propagation of the discharge plasma [38] as displayed in Fig 11(A). The generation areas of the discharge plasma are concentrated near the high-voltage electrode. The closer it is to the high-voltage electrode, the easier it is to excite the discharge plasma. Under water-covered condition, an electric field is present on the dielectric surface of the annular SDBD, regardless of the presence of water droplet. The electric field intensity is around along the external edge of the water droplet, the electric field intensities at both ends of the water droplet, near and far from the internal edge of the high-voltage electrode, are slightly higher. However, both values are smaller than those of the high-voltage electrode.

The change trend of the electric field distribution in the *z* direction with changes in *r* was calculated by studying the distribution of the external electric field on the surface of the annular SDBD by Li et al. [25]. The distribution of the external electric field along the *y* axis at the water-covered position was further studied. The values of *z* and *r* are 0.1–0.7 mm and 0–10 mm, respectively, along the *y* axis, where *r* is the distance from the circle center to the internal edge of the high-voltage electrode. A curve depicting the changes in the external electric field with *r* for different *z* values is shown in Fig 11(B), where the water droplet-covered position is marked by the blue semicircle in the figure. As Fig 11(B) shows, the external electric field amplitude near the high-voltage electrode can reach approximately 150 kV/cm, which is similar to the results reported by Jiang et al. [27]. The external electric field ***E***_ex_ close to the lower end of the high-voltage electrode decreases with increasing *z* and decreased to 30 kV/cm when *z* reaches 0.7 mm. The electric field intensity at the lower end increases sharply and slightly with an increase in *z*. It reaches the maximum value at *z* = 0.2 mm and is higher than ***E***_ex_ at the internal edge of the high-voltage electrode when *z* varies from 0.3 to 0.7 mm. Therefore, the micro-discharge channel is the brightest in the discharge images when developing to the lower end, which is similar to the conclusions of Zhu et al. [39]. The electric field intensity at the upper end also increased slightly and gradually weakens with decreasing *r*. This corresponds to the attenuation of the brightness of the discharge channel generated at the upper end along the discharge direction. The electric field intensity at the upper end reaches a maximum at z = 0.1 mm; The electric field strength here has reached 30 kV/cm. In practice, the critical electric field intensity for corona generation in air is 30 kV/cm [40]. Micro-discharge channels could be formed at the upper end in the experiments. This indicates that the relative permittivity of water droplets is significantly higher than those of air and dielectric surfaces, resulting in a local concentration at the three-phase boundary of the dielectric surface, water droplets, and air.

In this study, the electric field intensity of the annular SDBD surface gradually increases with an increase in *r* under water-covered condition. It is the largest at the internal edge of the high-voltage electrode, and the electric field intensity at both the upper and lower ends increases. The water droplet acts as a "secondary electrode" during the discharge.

### 4.2 Discharge theory of SDBD under water-covered conditions

**Fig 12:**
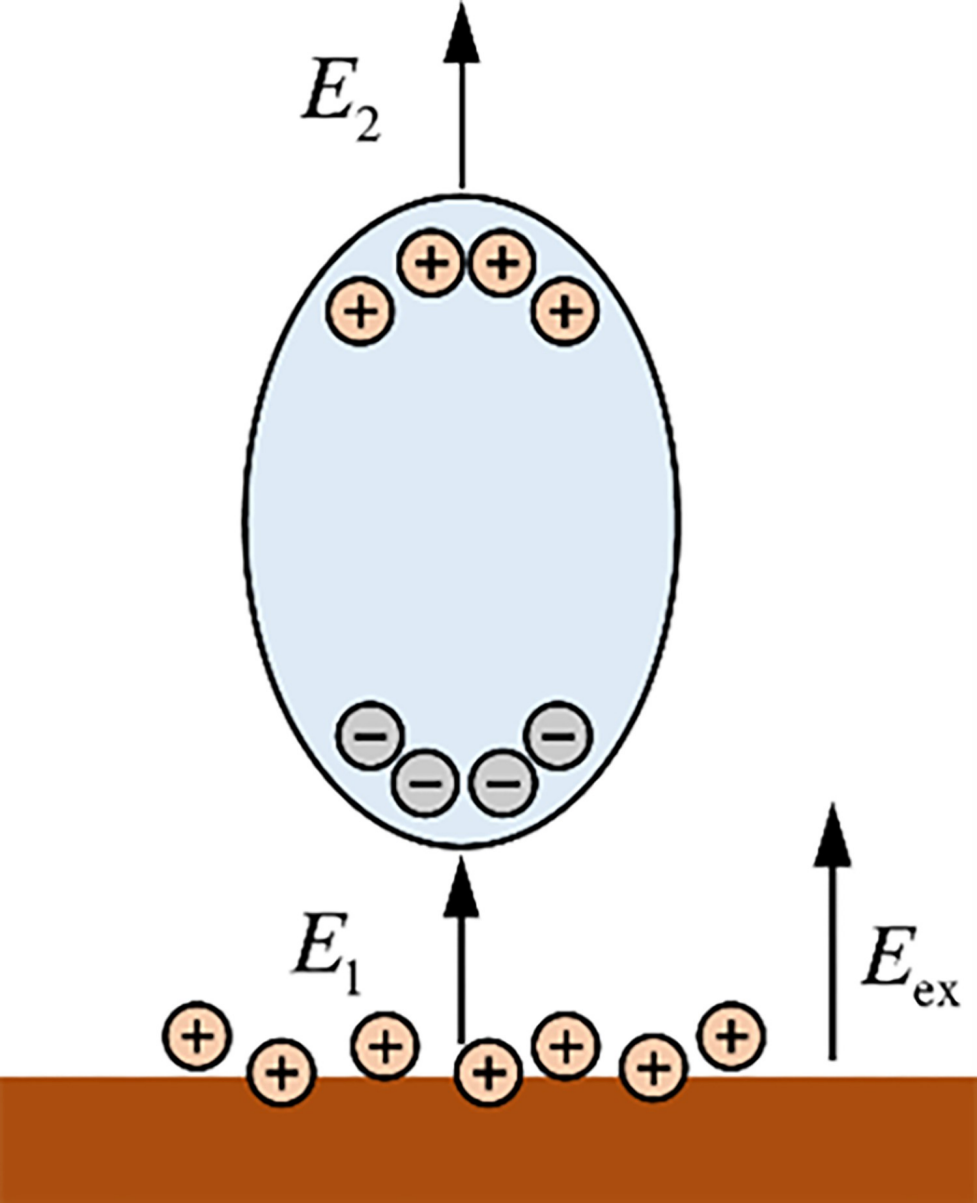
Schematic diagram of electric field generated by water droplet polarization.

For further analyzing the role of water droplet in the SDBD process, water is considered a dielectric in this study, and water is polarized in an electric field to generate polarization charges, as shown in Fig 12. The electric dipole moment of the water droplet is deflected under the action of the electric field ***E***_ex_. The interior of the water droplet finally presents a distribution state of negative electricity at one end and positive electricity at the other end. Therefore, many negative charges accumulate at the lower end [41]. The electric field of the SDBD is generated using an annular copper electrode. In this study, an induced electric field ***E***_1_ is present between the lower end and internal edge of the high-voltage electrode after the water droplet was polarized. This is conducive to the ionization and excitation of gases. Therefore, in the first stage of discharging from the high-voltage electrode to the lower end, a more intense and brighter discrete micro-discharge channel is generated.

Additionally, as the micro-discharge channel develops toward the lower end, the electrons in the plasma contact the water boundary, resulting in the electrolytic reaction of water [42, 43]. Chen et al. also found that water undergoes different chemical reactions with plasma in their study of the interaction between plasma and water solutions [44]. The chemical bonds of water molecules are broken owing to the abovementioned chemical reactions, and many free ions, such as H^+^, O^-^ and OH^-^, are present in the water droplet. Moreover, the reaction between plasma and water solution produces H_2_O^+^, NO_3_^-^ and other ions at the gas and liquid interface layer [10]. These liberty ions strengthen the electric field ***E***_1_. Similarly, numerous positive charges accumulate around the upper end, forming an electric potential difference between the ground electrode. The electric field ***E***_2_ is directed from the upper end of the dielectric surface to the center. This also illustrates that polarized water droplet can be regarded as a group of electric dipoles and is also the field source that generates part of the electric field [45], which confirms that water droplet plays a similar role as a "secondary electrode.”

Yan et al. [46] divided the charge accumulation in the dielectric barrier discharge process into two parts. A part of the charge accumulation is concentrated at the internal edge of the high-voltage electrode. The other part of the charge accumulates on the dielectric surface with the channel of the gas discharge plasma, and the existence of water droplet affects charge accumulation in this part under the water-covered condition in this study.

**Fig 13:**
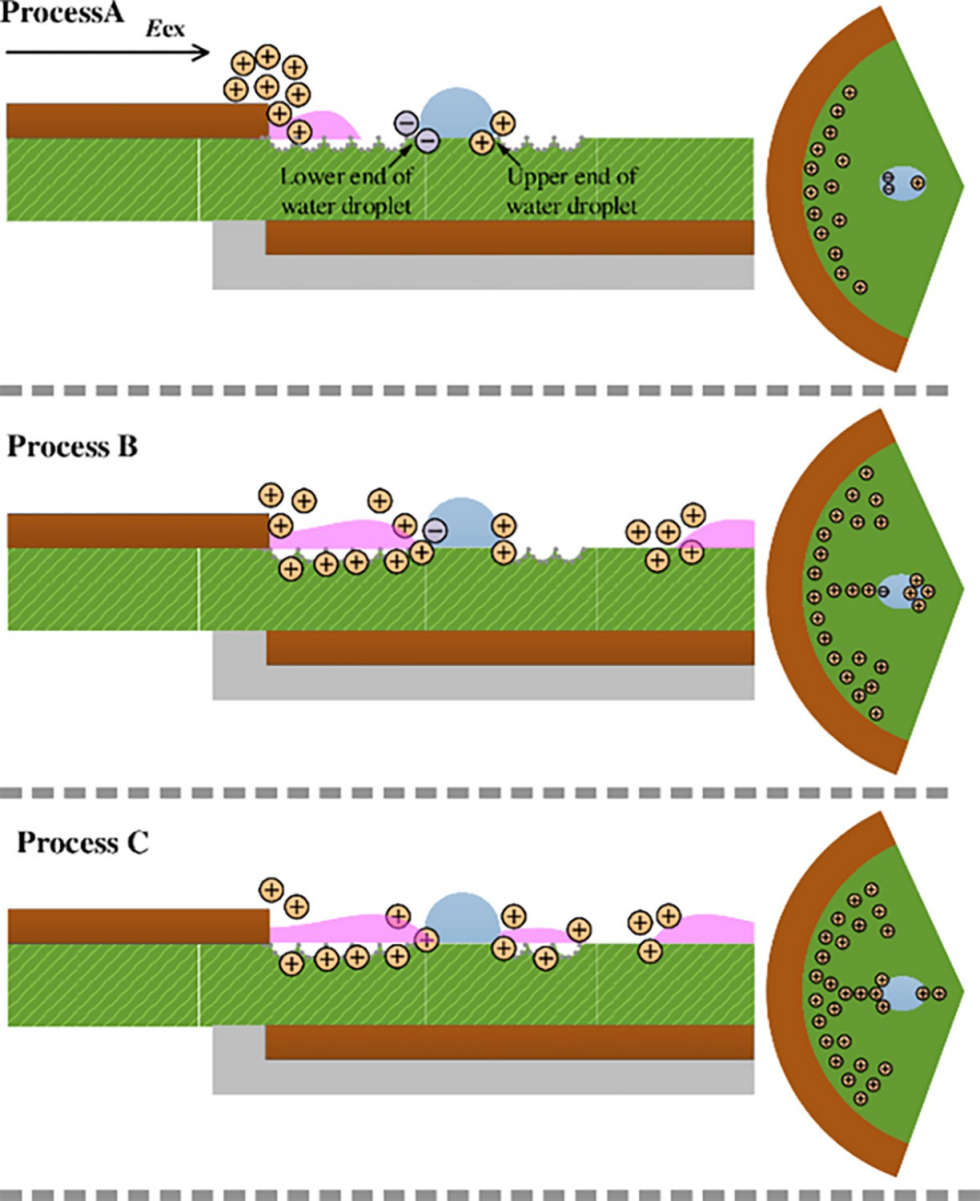
Schematic diagram of the discharge development process.

With an increase in the excitation voltage, the number of electron collisions in the air gap increases; the electron avalanche process intensifies, and finally, bright micro-discharge channels are formed, which can be observed in the discharge images. Charges mainly accumulate at the internal edge of the high-voltage electrode at a low-voltage amplitude, as shown in Fig 13. In process A, the electric field strength on the dielectric surface increases continuously with an increase in the voltage amplitude, which eventually leads to the breakdown of the surface gas, and discharges at the edge of the high-voltage electrode. The water droplet on the dielectric surface is polarized in the electric field and accumulates negative charges at the lower end, causing the internal edge of the high-voltage electrode to induce more positive charges. The gas breakdown in process A is strengthened by the increase in the voltage and accretion of positive charges. Therefore, more positive ions accumulate on the dielectric surface and move toward the water droplet under the action of the electric field force. Consequently, discrete micro-discharge channels, which are bright and different in length, develop along the dielectric surface and form the most intense channels at the internal edge of the high-voltage electrode and the lower end, as displayed in the discharge images. Based on this finding, high-density charges are mainly concentrated on the dielectric surface at the lower end. With the development of the micro-discharge channel toward the water droplet, the phenomenon of charge accumulation is more prominent. As the discharge enters process B, the chemical bonds of water molecules are broken, and abundant liberty ions exist in the water droplets after the micro-discharge channel contacts with water droplet. The negative ions are neutralized by the positive ions of the micro-discharge channels, and the excess positive ions accumulate at the lower end owing to the dielectric properties of the high-density water droplets [22]. A high resistance is observed as the charge moves forward. The electric field strength inside the water droplet is very low, and it cannot provide a sufficient electric field force to the positive ions; therefore, the speed of the movement of the positive ions reduces. Consequently, the water droplets cannot enter the micro-discharge channel.

In process C, polar water molecules are excited by a strong electric field, and field ionization occurs along the direction of the electric field. A large number of positive charges accumulate on the dielectric surface at the upper end, leading to a local electric field distortion. With the accumulation of positive charges on the water droplet, the electric field strength increases continuously. Finally, the gas at the front of the water droplet on the dielectric surface breaks down. The water droplets act as "secondary electrodes" to form discrete micro-discharge channels along the dielectric surface. Under water-covered condition, the charges of the annular SDBD accumulate on the dielectric surface and at the end of the water droplet. Shallow traps [47] on the surface of dielectrics have a strong ability to capture charged particles. Charged particles generated by water droplet polarization, discharge or the interaction between the discharge channel and the water droplet diffuse to the dielectric surface under the action of an external electric field. Water droplet adsorbs charged particles, leading to the deposition of charged particles around them. When the charged particles generated by the water droplet polarization accumulate to reach a certain threshold, the electric field in the air gap space changes, thereby affecting the discharge.

## 5. Conclusions

In this study, the discharge development process is demonstrated, and the effect of water droplet on discharge is studied through experimental research and simulation calculations of annular SDBD under water-covered condition. The following conclusions are drawn from this study.

1. The SDBD in the water-covered region is divided into two stages when the dielectric surface is covered with a water droplet. In the first stage, the high-voltage electrode generates a violent discrete channel that develops toward the lower end of the water droplet. The micro-discharge channel at the upper end of the water droplet develops into a dielectric surface in the second stage. The amplitude of the current pulse decreases under water-covered condition. The electromagnetic interference of the SDBD actuator will also be reduced.
2. As the conductivity of the droplet increases, the discharge intensity increases and the uniformity decreases, and the discharge gradually becomes dominated by discrete channels. The increase in droplet volume and conductivity will lead to a decrease in the discharge initial voltage.
3. Electric field distortion occurs at the three-phase boundary of the water droplet, dielectric surface and air under the water-covered condition of the SDBD. The electric field strength at the upper end of the water droplet can reach the air breakdown field strength 30 kV/cm, which excites air discharge and forms discrete micro-discharge channels with *d* = 2 mm.
4. Polarized water droplets can be used as a field source to form part of an electric field. Considering the analysis of gray image data, water droplet is considered to act as a "secondary electrode" during the discharge process. Water droplets affect the surface charge accumulation of the SDBD under water-covered condition, thereby affecting the development process of the micro-discharge channel. The discharge extends on the dielectric surface under the action of a water droplet, and the range of discharge increases for the SDBD.
